# Tris{2-[(2-amino­benzyl­idene)amino]­ethyl}amine

**DOI:** 10.1107/S1600536810043783

**Published:** 2010-11-06

**Authors:** Mariana Elizondo García, Sylvain Bernès, Nancy Pérez Rodríguez, Perla Elizondo Martínez

**Affiliations:** a Laboratorio de Química Industrial, CELAES, Facultad de Ciencias Químicas, UANL, Pedro de Alba s/n, 66451 San Nicolás de los Garza, NL, Mexico; b DEP Facultad de Ciencias Químicas, UANL, Guerrero y Progreso S/N, Col. Treviño, 64570 Monterrey, NL, Mexico

## Abstract

The title Schiff base, C_27_H_33_N_7_, is a tripodal amine displaying *C*_3_ symmetry, with the central tertiary N atom lying on the threefold crystallographic axis. The N—CH_2_—CH_2_—N conformation of the pendant arms is *gauche* [torsion angle = 76.1 (3)°], which results in a claw-like mol­ecule, with the terminal aniline groups wrapped around the symmetry axis. The lone pair of the apical N atom is clearly oriented inwards towards the cavity, and should thus be chemically inactive. The amine NH_2_ substituents lie in the plane of the benzene ring to which they are bonded. With such an arrangement, one amine H atom forms an *S*(6) motif through a weak N—H⋯N hydrogen bond with the imine N atom, while the other is engaged in an inter­molecular N—H⋯π contact involving the benzene ring of a neighbouring mol­ecule related by inversion. The benzene rings also participate in an intra­molecular C—H⋯π contact of similar strength. In the crystal structure, mol­ecules are separated by empty voids (*ca* 5% of the crystal volume), although the crystal seems to be unsolvated.

## Related literature

For applications of polyamines as metal extracta­nts, see: Wenzel (2008 ▶); Bernier *et al.* (2009 ▶); Galbraith *et al.* (2006 ▶). For other applications, see: Zibaseresht & Hartshorn (2005 ▶); Mercs *et al.* (2008 ▶). For similar *C*_3_ tripodal structures, see: Weibel *et al.* (2002 ▶); Işıklan *et al.* (2010 ▶); McKee *et al.* (2006 ▶); Glidewell *et al.* (2005 ▶). The software used for analysis of the empty voids in the crystal structure was SQUEEZE in *PLATON* (Spek, 2009 ▶).
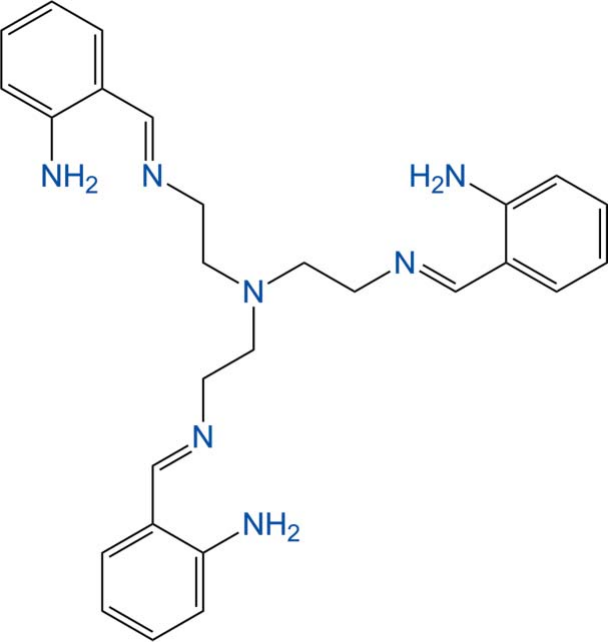


## Experimental

### 

#### Crystal data

C_27_H_33_N_7_
*M* *_r_* = 455.60
Trigonal, 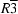

*a* = 13.1075 (18) Å
*c* = 25.985 (6) Å
*V* = 3866.3 (12) Å^3^
*Z* = 6
Mo *K*α radiation
μ = 0.07 mm^−1^
*T* = 300 K
0.40 × 0.40 × 0.18 mm

#### Data collection

Siemens *P*4 diffractometer
6668 measured reflections
1507 independent reflections
838 reflections with *I* > 2σ(*I*)
*R*_int_ = 0.033
2 standard reflections every 98 reflections  intensity decay: 2%

#### Refinement

*R*[*F* ^2^ > 2σ(*F* ^2^)] = 0.058
*wR*(*F* ^2^) = 0.176
*S* = 1.81
1507 reflections
110 parameters
H atoms treated by a mixture of independent and constrained refinement
Δρ_max_ = 0.51 e Å^−3^
Δρ_min_ = −0.21 e Å^−3^

### 

Data collection: *XSCANS* (Siemens, 1996 ▶); cell refinement: *XSCANS*; data reduction: *XSCANS*; program(s) used to solve structure: *SHELXS97* (Sheldrick, 2008 ▶); program(s) used to refine structure: *SHELXL97* (Sheldrick, 2008 ▶); molecular graphics: *Mercury* (Macrae *et al.*, 2006 ▶); software used to prepare material for publication: *SHELXL97*.

## Supplementary Material

Crystal structure: contains datablocks I, global. DOI: 10.1107/S1600536810043783/bq2243sup1.cif

Structure factors: contains datablocks I. DOI: 10.1107/S1600536810043783/bq2243Isup2.hkl

Additional supplementary materials:  crystallographic information; 3D view; checkCIF report

## Figures and Tables

**Table 1:** Hydrogen-bond geometry (Å, °) *Cg* is the centroid of the benzene ring.

*D*—H⋯*A*	*D*—H	H⋯*A*	*D*⋯*A*	*D*—H⋯*A*
N12—H12*A*⋯N4	0.92 (3)	2.02 (3)	2.700 (3)	129 (2)
N12—H12*B*⋯*Cg*^i^	0.86 (3)	2.70 (3)	3.430 (2)	143 (3)
C7—H7*A*⋯*Cg*^ii^	0.93	2.71	3.494 (3)	143

Symmetry codes: (i) 
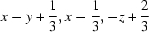
; (ii) 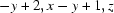.

**Fig. 1.**
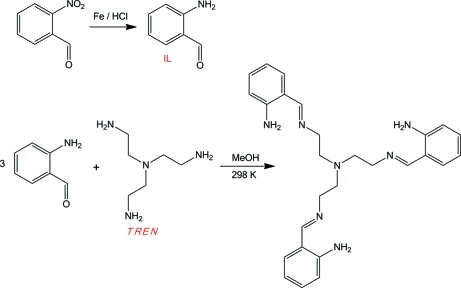
Synthetic route for the title compound.

**Fig. 2.**
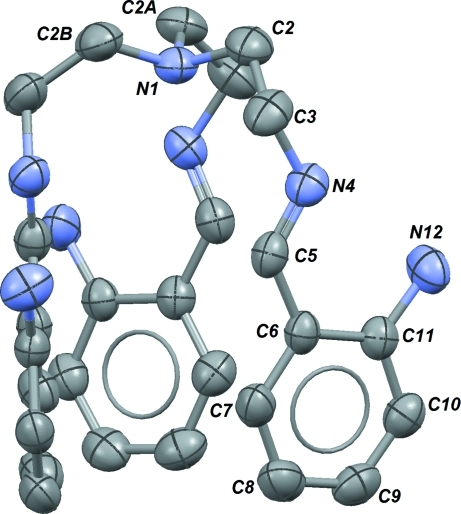
ORTEP-like view of the title molecule, with displacement ellipsoids at the 30% probability level. H atoms have been omitted for clarity, and only the asymmetric unit is completely labeled. Other atoms are generated by symmetry codes A: 2 - y, 1 + x - y, z and B: 1 - x + y, 2 - x, z.

## supplementary crystallographic information

### Comment

Recently, the research line of receptors with the ability to extract metal salts has grown in relevance, because of the harmful effects that anions and cations have in health and the environment. A class of such receptors includes polyamines, in which cations and anions are found in separate sites in a zwitterionic form of the ligand. As a consequence, the efficiency for solvent extraction of metal salts may be modulated trough pH adjustment (Wenzel, 2008). In these compounds, the metal ion coordinates in the deprotonated moiety, while the anion is associated to the protonated pendant groups (Bernier *et al.*, 2009; Galbraith *et al.*, 2006). The Schiff base condensation is a useful route to obtain polyamines including suitable structural characteristics in order to act as polytopic ligands. Some recent reports highlighted important applications of this type of compounds (Zibaseresht & Hartshorn, 2005; Mercs *et al.*, 2008).

We report herein on the synthesis (Fig. 1) and crystal structure of a new Schiff base, which, we hope, will allow to bond both cations and anions, depending on the pH. The molecule (Fig. 2) is a tripodal tertiary amine N*R*_3_ where *R* contains imine functionality. The tripodal N atom is placed on a 3-fold axis in a trigonal cell (*C*_3_ point symmetry). The pendant arms *R* are *gauche*, as reflected by torsion angle N1—C2—C3—N4, 76.1 (3)°, and the lone pair on N1 is directed toward the cavity formed by the arms. Similar arrangements giving claw-like molecules were observed in related tertiary amines, although in less symmetric Laue groups (*e.g.* Weibel *et al.*, 2002; Işıklan *et al.*, 2010). In some instances, closely related tripodal N*R*_3_ molecules approximate the *C*_3_ symmetry but with *R* arms lying in a plane rather than forming a closed cavity (McKee *et al.*, 2006). Glidewell *et al.* (2005) showed that the molecular conformation for this class of amines is determined mainly by direction-specific intra- and intermolecular interactions. In the case of the title amine, NH_2_ groups in the aniline moieties are engaged in both intra and intermolecular interactions: H12A forms a weak hydrogen bond with the imine atom N4, while H12B affords an intermolecular N—H···π contact, also of limited strength. The last significant contact is intramolecular: the C7—H7 aromatic group gives a C—H···π contact with the next arm in the molecule.

As mentioned, all non bonding contacts are rather weak. As a consequence, molecules are not densely packed in the crystal, and voids of *ca* 60 Å^3^ are available for solvent insertion. However, attempts to include non-diffracting solvent in the structural model using *SQUEEZE* (Spek, 2009) were unsuccessful. The chemical formula was thus left as unsolvated.

### Experimental

To a dissolution of 2-nitrobenzaldehyde (0.020 mol) in ethanol (60 ml), were added 11.114 g (0.20 mol) of iron, 90 µl of hydrochloric acid and 15 ml of distilled water. Immediately the mixture was refluxed for 90 min. The mixture was filtered off using Hyfo supercell, and the solvent was distilled, affording a yellow oil (Fig. 1, IL). In order to obtain the title molecule (I), a dissolution of 2.414 g of IL in 20 ml of methanol and 1060 µl of tris(2-aminoethyl)amine (*TREN*) were stirred at room temperature for 30 min, affording a yellow solid, (I), which was filtered off and recrystallized from acetonitrile. Suitable crystals were obtained as pale-yellow blocks by slow evaporation of an acetone solution at 298 K. m.p. 416–417 K; analysis found (calc. for C_27_H_33_N_7_): C 71.02 (71.18%), H 7.82 (7.30%), N 22.40 (21.52%); IR RTA: 3437, 3237 (NH ν_as_ and ν_s_), 1635 (C═N δ_s_), 1588 (NH δ_s_), 749 cm^-1^ (NH δ_s_). ^1^H NMR (200 MHz, CDCl_3_): δ, p.p.m.: 2.92 (6H, *t*, H_2_C—N), 3.69 (6H, *t*, H_2_C═N), 6.34 (6H, *s*, H_2_NAr), 6.62 (6H, *c*, Ar), 6.88 (3H, *dd*, Ar), 7.12 (3H, *td*, Ar), 8.17 (3H, *s*, Ar).

### Refinement

Amine H atoms H12A and H12B were found in a difference map and refined with free coordinates. Other H atoms were placed in idealized positions and refined as riding to their parent C atoms, with bond lengths fixed to 0.97 (methylene) or 0.93 Å (aromatic). Isotropic displacement parameters for H atoms were calculated as *U*_iso_(H) = 1.2*U*_eq_(carrier atom). A set of 21 reflections with *F*_o_ << *F*_c_ (probably because of a diffractometer instability) were omitted in least-squares refinement.

### Crystal data

**Table:**

C_27_H_33_N_7_	*D*_x_ = 1.174 Mg m^−^^3^
*M**_r_* = 455.60	Melting point: 416 K
Trigonal, *R*3	Mo *K*α radiation, λ = 0.71073 Å
Hall symbol: -R 3	Cell parameters from 70 reflections
*a* = 13.1075 (18) Å	θ = 4.8–12.3°
*c* = 25.985 (6) Å	µ = 0.07 mm^−^^1^
*V* = 3866.3 (12) Å^3^	*T* = 300 K
*Z* = 6	Prism, yellow
*F*(000) = 1464	0.40 × 0.40 × 0.18 mm

### Data collection

**Table:**

Siemens P4 diffractometer	*R*_int_ = 0.033
Radiation source: fine-focus sealed tube	θ_max_ = 25.0°, θ_min_ = 2.0°
graphite	*h* = −13→15
ω scans	*k* = −15→15
6668 measured reflections	*l* = −30→30
1507 independent reflections	2 standard reflections every 98 reflections
838 reflections with *I* > 2σ(*I*)	intensity decay: 2%

### Refinement

**Table:**

Refinement on *F*^2^	Secondary atom site location: difference Fourier map
Least-squares matrix: full	Hydrogen site location: inferred from neighbouring sites
*R*[*F*^2^ > 2σ(*F*^2^)] = 0.058	H atoms treated by a mixture of independent and constrained refinement
*wR*(*F*^2^) = 0.176	*w* = 1/[σ^2^(*F*_o_^2^) + (0.05*P*)^2^] where *P* = (*F*_o_^2^ + 2*F*_c_^2^)/3
*S* = 1.81	(Δ/σ)_max_ < 0.001
1507 reflections	Δρ_max_ = 0.51 e Å^−^^3^
110 parameters	Δρ_min_ = −0.21 e Å^−^^3^
0 restraints	Extinction correction: *SHELXL97* (Sheldrick, 2008), Fc^*^=kFc[1+0.001xFc^2^λ^3^/sin(2θ)]^-1/4^
0 constraints	Extinction coefficient: 0.0057 (9)
Primary atom site location: structure-invariant direct methods	

### Fractional atomic coordinates and isotropic or equivalent isotropic displacement parameters (Å^2^)

**Table:**

	*x*	*y*	*z*	*U*_iso_*/*U*_eq_	
N1	1.0000	1.0000	0.17108 (11)	0.0773 (10)	
C2	0.9753 (3)	0.8840 (2)	0.15415 (9)	0.0952 (9)	
H2A	1.0068	0.8904	0.1198	0.114*	
H2B	0.8906	0.8322	0.1524	0.114*	
C3	1.0266 (3)	0.8305 (3)	0.18909 (10)	0.0990 (10)	
H3A	1.0287	0.7663	0.1714	0.119*	
H3B	1.1069	0.8892	0.1978	0.119*	
N4	0.95737 (19)	0.78635 (19)	0.23590 (8)	0.0821 (7)	
C5	1.0077 (2)	0.8295 (2)	0.27831 (10)	0.0741 (7)	
H5A	1.0862	0.8891	0.2776	0.089*	
C6	0.9507 (2)	0.79175 (19)	0.32820 (9)	0.0680 (7)	
C7	1.0149 (2)	0.8459 (2)	0.37218 (10)	0.0825 (8)	
H7A	1.0933	0.9045	0.3688	0.099*	
C8	0.9669 (3)	0.8163 (3)	0.42032 (11)	0.0985 (9)	
H8A	1.0120	0.8540	0.4492	0.118*	
C9	0.8505 (3)	0.7297 (3)	0.42536 (10)	0.0915 (9)	
H9A	0.8165	0.7096	0.4579	0.110*	
C10	0.7853 (3)	0.6738 (2)	0.38354 (10)	0.0820 (8)	
H10A	0.7073	0.6147	0.3879	0.098*	
C11	0.8319 (2)	0.7026 (2)	0.33419 (9)	0.0696 (7)	
N12	0.7651 (2)	0.6450 (2)	0.29267 (9)	0.0958 (8)	
H12A	0.792 (3)	0.670 (3)	0.2599 (10)	0.115*	
H12B	0.692 (3)	0.597 (3)	0.2993 (11)	0.115*	

### Atomic displacement parameters (Å^2^)

**Table:**

	*U*^11^	*U*^22^	*U*^33^	*U*^12^	*U*^13^	*U*^23^
N1	0.0863 (15)	0.0863 (15)	0.0592 (18)	0.0432 (8)	0.000	0.000
C2	0.116 (2)	0.101 (2)	0.0686 (14)	0.0545 (18)	0.0064 (14)	−0.0093 (14)
C3	0.118 (2)	0.098 (2)	0.0928 (18)	0.0631 (19)	0.0324 (16)	0.0059 (15)
N4	0.0836 (15)	0.0817 (14)	0.0848 (14)	0.0442 (12)	0.0142 (12)	0.0027 (11)
C5	0.0668 (15)	0.0620 (14)	0.0941 (17)	0.0327 (12)	0.0095 (13)	0.0063 (13)
C6	0.0648 (15)	0.0560 (13)	0.0840 (16)	0.0307 (12)	0.0009 (12)	0.0046 (11)
C7	0.0821 (17)	0.0684 (16)	0.0913 (18)	0.0333 (14)	−0.0097 (14)	0.0050 (13)
C8	0.121 (3)	0.094 (2)	0.0859 (18)	0.058 (2)	−0.0204 (18)	−0.0012 (16)
C9	0.112 (2)	0.094 (2)	0.0842 (18)	0.063 (2)	0.0122 (16)	0.0217 (16)
C10	0.0820 (17)	0.0778 (17)	0.0947 (18)	0.0463 (14)	0.0110 (15)	0.0148 (14)
C11	0.0679 (15)	0.0647 (14)	0.0832 (15)	0.0384 (13)	0.0025 (13)	−0.0001 (13)
N12	0.0638 (14)	0.1025 (18)	0.1008 (16)	0.0263 (13)	0.0001 (13)	−0.0157 (14)

### Geometric parameters (Å, °)

**Table:**

N1—C2	1.455 (3)	C6—C11	1.413 (3)
N1—C2^i^	1.455 (3)	C7—C8	1.366 (4)
N1—C2^ii^	1.455 (3)	C7—H7A	0.9300
C2—C3	1.498 (4)	C8—C9	1.379 (4)
C2—H2A	0.9700	C8—H8A	0.9300
C2—H2B	0.9700	C9—C10	1.350 (4)
C3—N4	1.454 (3)	C9—H9A	0.9300
C3—H3A	0.9700	C10—C11	1.389 (3)
C3—H3B	0.9700	C10—H10A	0.9300
N4—C5	1.264 (3)	C11—N12	1.356 (3)
C5—C6	1.454 (3)	N12—H12A	0.92 (3)
C5—H5A	0.9300	N12—H12B	0.86 (3)
C6—C7	1.386 (3)		
			
C2—N1—C2^i^	111.28 (14)	C7—C6—C5	119.0 (2)
C2—N1—C2^ii^	111.28 (14)	C11—C6—C5	123.1 (2)
C2^i^—N1—C2^ii^	111.28 (14)	C8—C7—C6	122.3 (3)
N1—C2—C3	112.9 (2)	C8—C7—H7A	118.8
N1—C2—H2A	109.0	C6—C7—H7A	118.8
C3—C2—H2A	109.0	C7—C8—C9	118.9 (3)
N1—C2—H2B	109.0	C7—C8—H8A	120.5
C3—C2—H2B	109.0	C9—C8—H8A	120.5
H2A—C2—H2B	107.8	C10—C9—C8	120.7 (3)
N4—C3—C2	110.9 (2)	C10—C9—H9A	119.7
N4—C3—H3A	109.5	C8—C9—H9A	119.7
C2—C3—H3A	109.5	C9—C10—C11	121.5 (3)
N4—C3—H3B	109.5	C9—C10—H10A	119.2
C2—C3—H3B	109.5	C11—C10—H10A	119.2
H3A—C3—H3B	108.1	N12—C11—C10	120.6 (2)
C5—N4—C3	118.0 (2)	N12—C11—C6	120.7 (2)
N4—C5—C6	124.1 (2)	C10—C11—C6	118.7 (2)
N4—C5—H5A	117.9	C11—N12—H12A	120.7 (19)
C6—C5—H5A	117.9	C11—N12—H12B	115 (2)
C7—C6—C11	117.9 (2)	H12A—N12—H12B	123 (3)
			
C2^i^—N1—C2—C3	83.1 (3)	C6—C7—C8—C9	−0.1 (4)
C2^ii^—N1—C2—C3	−152.1 (3)	C7—C8—C9—C10	0.9 (4)
N1—C2—C3—N4	76.1 (3)	C8—C9—C10—C11	−1.1 (4)
C2—C3—N4—C5	−119.7 (3)	C9—C10—C11—N12	179.7 (2)
C3—N4—C5—C6	−178.2 (2)	C9—C10—C11—C6	0.6 (3)
N4—C5—C6—C7	−179.8 (2)	C7—C6—C11—N12	−178.9 (2)
N4—C5—C6—C11	−0.3 (4)	C5—C6—C11—N12	1.6 (3)
C11—C6—C7—C8	−0.3 (4)	C7—C6—C11—C10	0.1 (3)
C5—C6—C7—C8	179.1 (2)	C5—C6—C11—C10	−179.3 (2)

Symmetry codes: (i) −*y*+2, *x*−*y*+1, *z*; (ii) −*x*+*y*+1, −*x*+2, *z*.

### Hydrogen-bond geometry (Å, °)

**Table:**

*D*—H···*A*	*D*—H	H···*A*	*D*···*A*	*D*—H···*A*
N12—H12A···N4	0.92 (3)	2.02 (3)	2.700 (3)	129 (2)
N12—H12B···Cg^iii^	0.86 (3)	2.70 (3)	3.430 (2)	143 (3)
C7—H7A···Cg^i^	0.93	2.71	3.494 (3)	143

Symmetry codes: (iii) *x*−*y*+1/3, *x*−1/3, −*z*+2/3; (i) −*y*+2, *x*−*y*+1, *z*.
